# Correction: Tobollik et al. Burden of Disease Due to Traffic Noise in Germany. *Int. J. Environ. Res. Public Health* 2019, *16*, 2304

**DOI:** 10.3390/ijerph19158887

**Published:** 2022-07-22

**Authors:** Myriam Tobollik, Matthias Hintzsche, Jördis Wothge, Thomas Myck, Dietrich Plass

**Affiliations:** 1 German Environment Agency, Section Exposure Assessment and Environmental Health Indicators, Corrensplatz 1, 14195 Berlin, Germany; dietrich.plass@uba.de; 2 Department of Environment and Health, School of Public Health, Bielefeld University, Bielefeld, Universitätsstraße 25, 33615 Bielefeld, Germany; 3 German Environment Agency, Section Noise Abatement of Industrial Plants and Products, Noise Impact, Wörlitzer Platz 1, 06844 Dessau-Roßlau, Germany; matthias.hintzsche@uba.de (M.H.); joerdis.wothge@uba.de (J.W.); thomas.myck@uba.de (T.M.)

The authors wish to make the following corrections to this paper [1]:

## Error in Table

We found an error in our quantification of the population attributable fraction. It should be applied to the total population and not, as done in the paper, only to the exposed population.

Therefore, we assumed that the remaining German population is not exposed to noise levels above the respective counterfactual values, which results in 73,913,594 non-exposed people to road traffic noise, 81,502,094 non-exposed people to aircraft noise, and 75,923,594 non-exposed people to railway noise.

Accordingly, the results of the burden of disease quantification of ischemic heart disease attributable to traffic noise in Germany in 2016 changed as follows:

**Table 6:** Burden of disease due to ischemic heart disease attributable to traffic noise in Germany, 2016 (95% CI in brackets).

Health Outcomes	Road Traffic Noise		Aircraft Noise		Railway Noise	
	PAF	Life Years Lost	PAF	Life Years Lost	PAF	Life Years Lost
Morbidity	1.83% (0.65–2.93%)	7452 (2706–11,905) YLDs	0.08% (0.00–0.24%)	327 (0–976) YLDs	0.92% (0.00–2.8%)	3738 (0–11,402) YLDs
Mortality	0.42% (0.00–1.05%)	5345 (0–13,299) YLLs	0.05% (0.00–0.12%)	591 (0–1557) YLLs	–	–
Sum		12.797 (2706–25,204) DALYs		918 (0–2533) DALYs		3.738 (0–11.402) DALYs
Per 100,000 *		15.54 (3.29–30.61) DALYs		1.11 (0–3.08) DALYs		4.54 (0–13.85) DALYs

PAF = population attributable fraction, YLLs = years of life lost due to premature mortality, YLDs = years lived with disability, DALYs = disability-adjusted life years, IHD = ischemic heart disease, CI = confidence interval, * people exposed to the related traffic noise source.

**Table 9:** Sum of the DALYs attributable to traffic noise by evidence quality.

Health Outcomes	Road Traffic Noise	Aircraft Noise	Railway Noise
IHD morbidity	7452	327	3.738
Evidence	••	•	•
IHD mortality	5345	591	*
Evidence	•••	••	*
Annoyance	29,433	5669	23,367
Evidence	••	•••	•••
Sleep disturbance	24,654	3906	39,548
Evidence	•••	•••	•••
Sum (moderate evidence)	29,999	9575	62,915
Sum (low quality and better)	66,884	10,166	62,915
Sum (all)	66,884	10,493	66,653

IHD = ischemic heart disease, • = very low quality, •• = low quality, ••• = moderate quality, * no studies available.

## Text Correction

In Section 3.3. Combined Burden of the Different Health Outcomes:

“Considering all health outcomes, the total burden due to the different traffic noise sources is 66,884 DALYs for road traffic noise, 10,166 DALYs for aircraft noise, and 62,915 DALYs for railway noise (see Table 9). However, taking into consideration the evidence level of the risk-outcome-pairs, the burden of road traffic noise is much lower when restricting the health outcomes to those showing moderate evidence (29,999 DALYs). The burden due to aircraft noise exposure is estimated at 9575 DALYs when considering only moderate evidence because the association of aircraft noise and ischemic heart disease is valued with very low (morbidity) and low (mortality) evidence.”

Considering all included health outcomes regardless of the evidence of their association, the results indicate that the total burden of disease is 66,884 DALYs for road traffic noise, 10,166 DALYs for aircraft noise, and 62,915 DALYs for railway noise.

The authors would like to apologize for any inconvenience caused to the readers by these changes.
